# The Association Between Obstructive Sleep Apnea and Alzheimer’s Disease: A Meta-Analysis Perspective

**DOI:** 10.3389/fnagi.2016.00078

**Published:** 2016-04-12

**Authors:** Farnoosh Emamian, Habibolah Khazaie, Masoud Tahmasian, Guy D. Leschziner, Mary J. Morrell, Ging-Yuek R. Hsiung, Ivana Rosenzweig, Amir A. Sepehry

**Affiliations:** ^1^Sleep Disorders Research Center, Kermanshah University of Medical Sciences (KUMS)Kermanshah, Iran; ^2^Department of Psychiatry, University of Social Welfare and Rehabilitation SciencesTehran, Iran; ^3^Sleep Disorders Centre, Guy’s and St Thomas’ HospitalLondon, UK; ^4^Sleep and Brain Plasticity Centre, Department of Neuroimaging, Institute of Psychiatry, Psychology & Neuroscience (IOPPN), King’s College and Imperial CollegeLondon, UK; ^5^Academic Unit of Sleep and Breathing, National Heart and Lung Institute, Imperial College and NIHR Respiratory Disease Biomedical Research Unit at the Royal Brompton and Harefield NHS Foundation Trust and Imperial CollegeLondon, UK; ^6^Division of Neurology, Faculty of Medicine, University of British ColumbiaVancouver, BC, Canada

**Keywords:** obstructive sleep apnea, Alzheimer’s disease, meta-analysis, sleep-disordered breathing, prevalence

## Abstract

Alzheimer’s disease (AD) and obstructive sleep apnea (OSA) are highly prevalent, chronic conditions with intriguing, yet poorly understood epidemiological overlap. To date, the amount of OSA syndrome present in patients with AD across literature remains unknown. To address this question, we collected all available published clinical data and analyzed them through a quantitative meta-analytical approach. The results of our quantitative meta-analysis suggest that the aggregate odds ratio for OSA in AD vs. healthy control was 5.05 and homogeneous. This reflects that patients with AD have a five times higher chance of presenting with OSA than cognitively non-impaired individuals of similar age. Moreover, these data suggest that around half of patients with AD have experienced OSA at some point after their initial diagnosis. The additive impact of progressive changes in sleep quality and structure, changes in cerebral blood flow and the cellular redox status in OSA patients may all be contributing factors to cognitive decline and may further aggravate AD progression. It is hoped that the high OSA rate in AD patients, as suggested by the findings of our meta-analysis, might provide a sufficient clinical incentive to alert clinicians the importance of screening patients for OSA in AD, and stimulate further research in this area.

## Introduction

Alzheimer’s disease (AD) and obstructive sleep apnea (OSA) are highly prevalent, chronic conditions with intriguing, yet poorly understood epidemiological overlaps (Ancoli-Israel et al., 1991; Khazaie et al., 2011, 2013; Chan et al., 2013; Khazaie and Maroufi, 2014; Pan and Kastin, 2014; Reitz and Mayeux, 2014; Heinzer et al., 2015; Peter-Derex et al., 2015; Rosenzweig et al., 2015; Khaledi-Paveh et al., 2016). From a clinical perspective, it is important to know the prevalence of OSA in patients with AD, as well as the incidence of AD in patients with OSA, in order to plan resources and modify treatment for co-morbidities (Pan and Kastin, 2014; Osorio et al., 2015).

Preclinical studies, suggest a causal relationship between untreated OSA and instigation of neurodegenerative processes underlying AD, although the process is far from clear (Pan and Kastin, 2014; Rosenzweig et al., 2015). Nonetheless, the results of several studies suggest that OSA might be a reversible cause of cognitive impairment and dementia, and that treatment of OSA, particularly in the early stages of AD, when patients are still largely independent, may decelerate dementia progression (Ancoli-Israel et al., 2008; Cooke et al., 2009b; Troussière et al., 2014). Correlation between symptoms of OSA and cognitive impairment has been shown in patients with dementia (Ancoli-Israel and Coy, 1994). For example, a study that investigated AD patients in a nursing home setting has found that those with severe dementia had significantly more severe OSA compared to those with mild to moderate or no dementia, and that those with more severe OSA had significantly more severe dementia (Ancoli-Israel et al., 1991). Similarly, the gold standard treatment for OSA, continuous positive airway pressure (CPAP), has been shown to be partially effective in improving episodic verbal learning, memory, cognitive flexibility and mental processing speed in patients with co-morbid AD and OSA (Ancoli-Israel et al., 2008). In another preliminary study, long-term CPAP treatment was associated with a lasting improvement in sleep and mood, as well as a slowing of cognitive deterioration in AD (Cooke et al., 2009b). In a further study of patients with mild to moderate AD, CPAP was shown to result in deeper sleep after just one night, and these improvements were maintained for several weeks (Cooke et al., 2009a).

It has been reported that OSA may induce neuroinflammatory and neurotrophic changes in affected and susceptible patients, and OSA-induced brain injury has been demonstrated in animal experimental models (Xu et al., 2004; Lavie, 2015; Rosenzweig et al., 2015). In OSA, nocturnal episodes of complete or partial pharyngeal obstruction result in intermittent hypoxia, reoxygenation, and sleep fragmentation (Malhotra et al., 2015). Serious cardiovascular and metabolic co-morbidities can be associated with OSA (Malhotra et al., 2015), and they may independently contribute to, and further aggravate, progression and severity of AD (Bliwise, 2013). The additive impact of progressive changes in sleep quality and structure, changes in cerebral blood flow, neurovascular and neurotransmitter changes, and the cellular redox status and neural regulation in OSA patients may all constitute contributing factors to further cognitive decline, as well as to aggravate progression of AD (Rosenzweig et al., 2014, 2015; Veasey, 2014; Lavie, 2015).

To date, the magnitude of the OSA observed in patients with AD remains unknown, with only a few (likely underpowered studies) available in the literature (Bliwise, 2013). The aim of this study was to provide a more convergent answer regarding the presence of OSA in patients with AD. To achieve this aim, whilst recognizing the constraints of smaller single studies, we undertook to find and collect all available published clinical data across applicable studies, and to analyze it through a more powerful, quantitative meta-analytical approach.

## Materials and Methods

Following the Preferred Reporting Items for Systematic Reviews and Meta-Analyses (PRISMA) statement (Moher et al., 2009), search of medical literature via PubMed was carried out in July 2015 We used the following key terms: (“Sleep Apnea Syndromes” [Mesh] OR “Sleep Apnea, Obstructive” [Mesh] OR “Sleep Apnea”) AND (“AD” OR Alzheimer’s disease [Mesh]). Further studies were identified through reviewing studies and reference tracing of retrieved articles. We included any study reporting prevalence of OSA in AD. Exclusion criteria were as follows: (1) case reports, reviews, meta-analysis, or animal studies; (2) non-AD studies (e.g., studies on other dementia or mild cognitive impairment (MCI) patients); (3) non-OSA studies (e.g., studies on sleep fragmentation or central sleep apnea); (4) interventional studies (e.g., CPAP or treatment effects); and (5) duplicate material. The search of the literature was subsequently updated on December 2015 and Alzheimer’s Dementia was included in the list of key terms. No new study met our criteria to be included.

For each study, the total number of AD patients and number of AD patients with OSA was extracted. When possible, similarly this approach was implemented for healthy control subjects’ data. These data allowed us to calculate an odd ratio (OR) for each study and subsequently an aggregate measure using the Biostat’s statistical package (Borenstein et al., 2005). For the analysis, random effect model was selected *a priori*. The OR measurement allowed us to estimate the difference in occurrence of OSA in AD compared to healthy control. In the event of limited dataset, we have run sensitivity analysis with select moderating variables. By the same token, investigation of heterogeneity and publication bias was carried out when applicable. Each study provided sufficient data for sensitivity analysis on the AD patients including, gender, average age, MMSE score, and dementia rating. For all other selected variables, if less than three studies reported data, no analyses were undertaken.

## Results

### Description of the Study Selection

**Figure 1:**
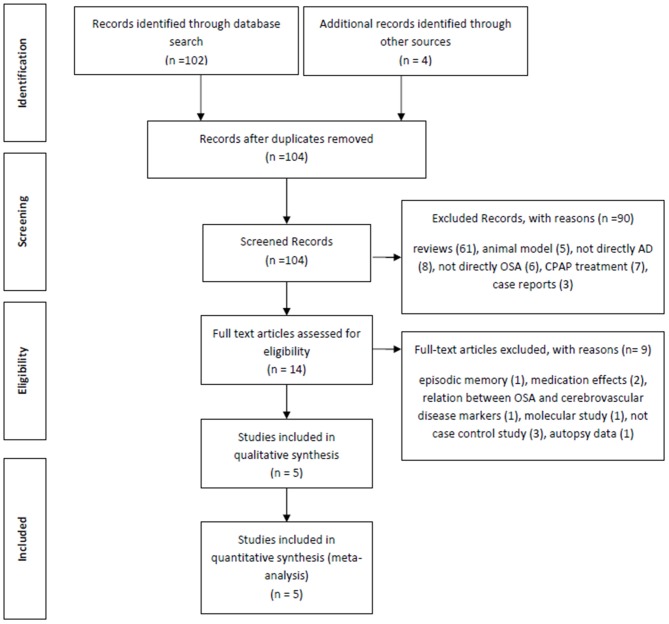
**Flow chart depicting article selection strategy**.

The initial electronic search from PubMed yielded 102 articles. In addition, four further manuscripts were found from other sources (see below). Of these 102 articles, 61 studies were reviews and were therefore excluded, a further five were excluded because they studied animal models, three studies were case reports, eight studies did not include any patients with Alzheimer’s Dementia, six studies did not consider any OSA patients, seven were about CPAP effects and a further seven were not deemed relevant (for example: two studies were on medication effects, one was about the relationship between OSA and cerebrovascular disease markers, two studies were not case-controls, one was a molecular study and the final excluded study was about autopsy data). Based on our predetermined exclusion criteria, the following four studies from the additional reference tracking were excluded: two were a duplication from our PubMed search (Reynolds et al., 1985; Hoch et al., 1986), one study was about episodic memory and not directly AD (Daurat et al., 2008) and the final study was not a case control study (Yaffe et al., 2011; Figure 1). The final summary of the five included cross-sectional studies (Smallwood et al., 1983; Reynolds et al., 1985, 1987; Hoch et al., 1986, 1989) is presented in Table 1.

**Table 1:** **Demographic representation of the included cross-sectional studies**.

Variables	Groups	Hoch et al. (1989)	Reynolds et al. (1987)	Hoch et al. (1986)	Reynolds et al. (1985)	Smallwood et al. (1983)
Number of subjects	Patients	15	15	24	21	15
	Controls	12	15	56	23	40
Number of males (%)	Patients	2 (13.33%)	0 (0%)	6 (25%)	6 (28.5%)	11 (73.3%)
	Controls	5 (41.6%)	3 (20%)	27 (48.21%)	9 (39.1%)	34 (85%)
Age (years)	Patients	74.5 ± 5.1	73.3 ± 9.1	71.5 ± 8.1	70.3 ± 7.9	65.5 ± 2.3 (male)
						69.5 ± 4.4 (female)
	Controls	70.2 ± 5.6	72.6 ± 7.8	69.3 ± 5.4	69.3 ± 5.6	Male (60 ± 1.31)
						Female (65.5 ± 2.2)
						Young male (25.2 ± 0.59)
BMI	Patients	22.8 ± 4.2	21.9 ± 4.7	20% of ideal	20% ideal	±15% of ideal
			(*n* = 12)	bodyweight	body weight	body weight
	Controls	27.2 ± 5.3	27.1 ± 5.0	20% of ideal	20% ideal	±15% of ideal
			(*n* = 13)	body weight	body weight	body weight
Education (years)	Patients	11.1 ± 4.3	–	11.3 ± 4.1	11.3 ± 4.1	–
	Controls	12.9 ± 3.7	–	14.8 ± 3.8	15.4 ± 4.1	–
Dementia diagnosis criteria	Patients	NICNDS-ADRDA, DSMIII	DSMIII	DSMIII	DSMIII	DSM III
Folstein mini-mental state score (MMSE)	Patients	17.2 ± 7.2	15.7 ± 8.7	18 ± 4.6	18.2 ± 4.9	–
	Controls	29.3 ± 0.6	29.2 ± 0.7	29.3 ± 0.8	29.3 ± 0.9	–
Hachinski ischemia score	Patients	1.0 ± 0.8	1.2 ± 1.3	1.1 ± 1.1	1.9 ± 1.3	–
	Controls	1.1 ± 0.8	1.2 ± 0.9	0.8 ± 0.7	1.1 ± 0.9	–
Dementia rating scale	Patients	10.7 ± 4.0	10.1 ± 5.8	8.6 ± 5.5	8.5 ± 4.8	–
OSA diagnosis method	Patients	PSG	24-chanel polygraphs (Grass 78B)	PSG	PSG	Respiratory inductive plethysmography
	Controls	PSG	24-chanel polygraphs (Grass 78B)	PSG	PSG	Respiratory inductive plethysmography
OSA %	Patients	53.33%	38%	41.66	42.85%	53.33%
	Controls	33.33%	13%	5.35%	4.34%	27.5%

*Note: OSA: obstructive sleep apnea; DSM: the Diagnostic and Statistical Manual of Mental Disorders; NICNDS-ADRDA: National Institute of Neurological and Communicative Disorders and Stroke and the Alzheimer’s Disease and Related Disorders Association; PSG: polysomnography*.

### Description of the Five Cross-Sectional Included Studies

In the included studies, numbers of patients ranged from 15 and 24, and healthy controls from 12 to 56. The majority of studies investigated female patients, with the exception of one study that reported on 73.3% male patients. The age of the patients ranged from 65.5 to 74.5 years across studies. AD was diagnosed predominantly according to Diagnostic and Statistical Manual of Mental Disorders (DSM-III) criteria (Kendell, 1980) for AD (4/5 studies) and only one study (Hoch et al., 1989) used both the DSM-III and the NINCDS-ADRDA Alzheimer’s Criteria, which were proposed in 1984 by the National Institute of Neurological and Communicative Disorders and Stroke and the AD and Related Disorders Association (McKhann et al., 1984) and; and four study explicitly specified probable AD. In terms of education, the groups were uneven in that controls on average were more educated than the patients; controls number of years of education ranged from 12.9 to 15.4 years vs. patients number of years of education ranged from 11.1 to 11.3. Folstein mini-mental state score was utilized in four out of five studies and it ranged from 15.7 to 18.2. Dementia Rating Scale was reported in three of four studies and ranged from 8.5 to 10.7. All data emerged from published studies with samples from the USA (Table 1). There was heterogeneity across studies in terms of how OSA was assessed; 3 of 5 studies used polysomnography, while others used either 24-chanel polygraph or respiratory inductive plethysmography.

### Effect Size, Heterogeneity, and Measurement of Bias

**Figure 2:**
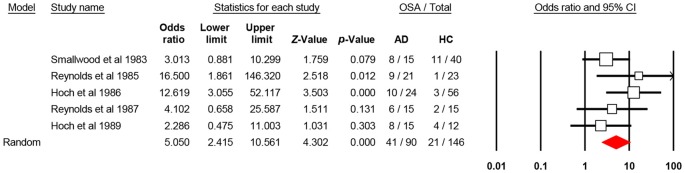
**Forest plot shows the aggregate comparison between patients with Alzheimer’s disease (AD) and healthy controls in terms of obstructive sleep apnea (OSA).** CI, confidence interval.

The aggregate odds ratio for the occurrence of OSA in AD vs. healthy control was 5.05 [*N* = 5; 95% confidence interval (CI): 2.41–10.56, *P*-value < 0.001; *Q*-value: 4.44; *P*-value: 0.35; I^2^: 9.85] and homogeneous. It is of note that only two of the five studies (Reynolds et al., 1985; Hoch et al., 1986) showed significant 95% CI, which may suggest that each study weights differently in the analysis. In fact, these two studies, have the largest sample in the AD study arm. With the broad 95% CI in mind and the level of significance (alpha 0.05), this effect size, odds ratio, suggests that OSA occurs 5.05 times more in AD than in healthy controls. For odds ratio analysis, of the 90 AD enrolled patients, 41 had OSA (~45%), and of the 146 healthy controls, 21 had OSA (~14%; Figure 2). This suggests that nearly 50% of the investigated AD patients experienced OSA at some time after diagnosis. For publication bias analysis, two quantitative statistics, the Begg and Mazumdar rank correlation for Kendall’s tau with and without continuity correction and Egger’s regression intercept were non-significant at alpha 0.05. The non-significant results for the Begg and Mazumdar, and Egger’s regression provide evidence to suggest the lack of publication bias with the current set of studies.

### Sensitivity Analysis

Single variable meta-regression using mixed effect (method of moments) with AD parameters, showed no significant effect of percent male [Point estimate: 0.006; *P*-value: 0.517; *N* = 5], average age [Point estimate: −0.056; *P*-value: 0.304; *N* = 5], MMSE score [Point estimate: 0.566; *P*-value: 0.230; *N* = 4], dementia rating scale score [Point estimate: −0.831; *P*-value: 0.063; *N* = 4], and Hachinski ischemia score [Point estimate: 1.521; *P*-value: 0.318; *N* = 4] on OSA odds of occurrence in AD patients vs. healthy control.

The OSA assessment approach affected the OR’s magnitude. For 3 out of 5 studies that used the PSG approach, the OR was 7.248, 95%CI ranged between 2.121 and 24.771, it was significant (*P*-value: 0.002), and homogeneous [*Q*-value: 3.201; *P*-value: 0.202; I^2^: 37.567]. For the other two studies, given the heterogeneity in the approach and number of studies for analysis, no further analysis was conducted. Similarly, the aggregate OR was larger for the studies explicitly reporting on probable AD [OR: 6.335; 95%CI: 2.540–15.803; *P*-value: 0.001, *N* = 4].

No further analysis could be done on the effect of BMI given the heterogeneity in the methods on reporting data and low number of studies. Given the ratio of the overall number of studies to number of variables examined, the sensitivity analyses data should be interpreted with caution.

## Discussion

The results of our meta-analysis suggest that patients with AD have five times higher chance of presenting with OSA compared to cognitively non-impaired individuals of similar age. Our data further suggest that in these studies, around 50% of patients with AD have experienced OSA at some time after their initial diagnosis.

Whilst our data cannot be taken to suggest causality or direction of the temporal relationship between two debilitating disorders, it certainly raises questions about the possible clinical risk of their additive impact. OSA has been shown to cause progressive central nervous changes and excessive daytime somnolence in susceptible patients, and this can contribute to further cognitive decline or AD progression (Rosenzweig et al., 2013, 2015). Any intervention that improves cognition in patients with dementia is likely to have broad impact, since improved daily function implies greater independence for the patient, less caregiver burden, fewer nursing service and social support needs, and generally reduced disease-associated costs (Cooke et al., 2009b; Bliwise, 2013). Currently there are only a few studies that explored the impact of treatment with CPAP of OSA on AD course, and some clinicians argue that the current evidence for cognitive benefits is not sufficient to dictate that treatment as imperative for all patients (Ancoli-Israel et al., 2008; Cooke et al., 2009a; Bliwise, 2013; Troussière et al., 2014; Osorio et al., 2015). Nonetheless, there appears to be evidence to suggest that CPAP is beneficial in treating hypersomnolence and sleep integrity in some patients with AD (Bliwise, 2013). Also, several studies over years have shown an indirect association between excessive daytime sleepiness and the development of cognitive decline (Jaussent et al., 2012; Keage et al., 2012). Additionally, beneficial effects of treatment of OSA, on cognitive performance, vigilance and excessive daytime sleepiness have been shown in recent meta-analyses (Marshall et al., 2006; Olaithe et al., 2014) and a meta review (Bucks et al., 2013). These data have recently been extended to show the beneficial effects of the CPAP therapy on excessive daytime sleepiness in older OSA patients (McMillan et al., 2014).

Fewer studies, have investigated the link between OSA and the onset of MCI and AD. A recent study of a well-characterized longitudinal cohort (the AD Neuroimaging Initiative (ADNI) cohort), has reported a significant association of sleep-disordered breathing with an earlier age at MCI and AD onset (Osorio et al., 2015). In this study, OSA appeared to confer risk of onset of AD, starting one decade earlier even when accounting for possible confounding factors such as sex, APOε4 status, diabetes, depression, BMI, cardiovascular disease, hypertension, age at baseline, and education of participants. Moreover, this link appeared significantly attenuated in those patients who used CPAP, suggesting that use of CPAP may delay progression, or onset, of MCI. The similar effect of CPAP on delay in age at AD onset was not demonstrated in this study (Osorio et al., 2015). Correspondingly, in an earlier study of 298 community-dwelling women, those with co-morbid OSA were shown as more likely to develop MCI or dementia at the 5-year follow-up (Yaffe et al., 2011). In the same way, another recent pilot study has demonstrated that CPAP treatment of severe OSA, in mild-to-moderate AD patients was associated with significantly slower cognitive decline over a 3-year follow-up period (Troussière et al., 2014). Taken together, these data emphasize the importance of detecting and treating OSA in this population.

Analogously, an accumulating body of clinical and pre-clinical research suggests that OSA might be one of rare modifiable factors in the pathomechanisms of AD (Bliwise, 2013; Pan and Kastin, 2014; Osorio et al., 2015; Rosenzweig et al., 2015). The association between OSA and cerebral spinal fluid (CSF) AD-biomarker changes including elevated CSF phosphorylated tau and β-amyloid 42 in elderly with the APOε3/3 alleles has been demonstrated (Osorio et al., 2014). By the same token, recent neuroimaging studies have suggested that sleep disruption, such as those occurring during OSA, can presents a mechanistic pathway through which β-amyloid pathology may contribute to hippocampus-dependent cognitive decline in the elderly (Mander et al., 2013, 2015). In addition, increasing evidence suggests that AD and OSA pathogenesis includes strong interactions with immunological mechanisms in the brain (Heneka et al., 2015a,b; Rosenzweig et al., 2015). Genome-wide analysis further suggest that several genes that increase the risk for sporadic AD also encode factors that regulate glial clearance of misfolded proteins and the inflammatory reaction (Heneka et al., 2015a) Comparably, abnormal protein folding and neuroinflammation have been also demonstrated or suggested by preclinical and clinical OSA studies (Roussel et al., 2013; Rosenzweig et al., 2015).

## Limitations

Given the strong association between the two disorders, the lack of epidemiological studies of co-morbid AD and OSA undertaken to date is notable; our literature search yielded only a few applicable studies, all conducted in the late 1980’s. Moreover, it is noteworthy that due to the clinical and cognitive presentations of the enrolled patients and the lack of reported neuroimaging or biomarkers data, we could potentially acknowledge that most of these patients were of probable AD. The small amount of available epidemiological data reflects the difficulties in conducting overnight studies in such a vulnerable patient population. Over the last two decades, however, there has been significant progress in the diagnosis of AD (Rhodius-Meester et al., 2015; Carmona et al., 2016; Salvatore et al., 2016), and it is possible that our results are not representative of the current socio-demographics, or indeed of the current AD cohort. It is arguable that prevalence could be even higher given that risks shared by both disorders (e.g., ageing and obesity) are on increase (Yaffe et al., 2014; Peter-Derex et al., 2015; Rosenzweig et al., 2015; Willette et al., 2015).

With this view, and keeping in mind the limitations of our study, several other findings from our data merit a further mention. In our study, healthy and otherwise well-matched controls were found to be significantly more educated than the patients. Previous studies have suggested that individuals with higher education and higher occupational attainment have a reduced risk of developing AD (Harris et al., 2015). Indeed, it has been suggested that higher cognitive reserve might protect against neurocognitive deficits induced by OSA (Rosenzweig et al., 2015) Also of note, in all but one study (Smallwood et al., 1983) women were in the majority, suggesting either higher prevalence, or as of yet unexplained recruitment bias on part of researchers conducting those studies. It is known that OSA is more prevalent in men than in women and that it increases with age and obesity (Franklin and Lindberg, 2015). The prevalence of OSA in women, however, is known to increase significantly after menopause (Koo et al., 2016). Similarly, the onset of menopause in women increases their risk of AD, possibly due to depletion of estrogens (Christensen and Pike, 2015). This may be of particular clinical importance, as the limited data available suggest that women with OSA may have greater risk of hypertension and endothelial dysfunction, and that they could be more likely to develop comorbid conditions and have increased mortality (Christensen and Pike, 2015). In addition, several authors have argued that both aging and menopause contribute to increasing levels of neuroinflammation, which, as mentioned, is increasingly recognized as one of the major pathomechanisms of AD and OSA (Christensen and Pike, 2015; Heneka et al., 2015a; Rosenzweig et al., 2015).

Due to the small number of original studies and their relatively small patient cohorts, our study was not sufficiently powered to investigate the effect of several important confounding factors such as APOε4 status, diabetes, depression, BMI, cardiovascular disease, hypertension. It is important to acknowledge that, although the type of OSA assessment affected the results, we did not examine the sensitivity of each approach. However, none of these factors have been shown to influence the efficacy of the CPAP treatment in AD. Nevertheless, it could be argued that they could, present a putative causal pathway for OSA and AD (Bliwise, 2013). Given all the limitations, caution should be used when interpreting our findings. To determine the interactions between OSA and its treatment on AD progression or severity, longitudinal epidemiological studies are needed that address these limitations.

## Conclusion

The accumulated impact of even small improvements in the quality of life, health and associated lower therapy costs for AD is likely to have enormous consequences when one considers that it is a chronic disorder refractory to treatment and that it carries a huge morbidity and mortality burden to patient and their carers, local communities, and overall society. Although currently there is no conclusive evidence that treating OSA in AD will have a major impact on AD course (Bliwise, 2013), several authors have argued that there is enough clinical evidence to suggest CPAP treatment may be at least beneficial in terms of alleviating daytime hypersomnolence, excessive napping and lethargy, all of which are recognized, and incapacitating problems in many AD patients (Bliwise, 2013). It has been further argued that, as long as there is a realistic appraisal of what goals of intervention should be expected, the treatment should be encouraged (Bliwise, 2013). Moreover, clinicians have proposed that in selected cases, treatment of OSA in AD patients may have dramatic effect; the data reviewed in our study appears to further support this empirical knowledge (Yaffe et al., 2011; Bliwise, 2013; Troussière et al., 2014; Osorio et al., 2015). It is hoped that the high occurrence of OSA in AD patients suggested by our meta-analyses might alert clinicians to importance of screening patients with AD for co-morbidities such as OSA. Future prospective studies are required to confirm this association in neuropathologically verified cases of AD, as well as to delineate distinct interplay of co-morbid OSA with different types of neurodegenerative processes (e.g., in vascular vs. mixed dementia vs. AD).

## Author Contributions

FE, HK, MT, IR, AAS designed the study; FE, MT collected the data; FE, AAS performed all analyses; FE, HK, GDL, MJM, G-YRH, IR, AAS wrote the manuscript. All authors contributed to writing of this manuscript.

## Conflict of Interest Statement

The authors declare that the research was conducted in the absence of any commercial or financial relationships that could be construed as a potential conflict of interest.
